# Recognition of duplex RNA by the deaminase domain of the RNA editing enzyme ADAR2

**DOI:** 10.1093/nar/gku1345

**Published:** 2015-01-06

**Authors:** Kelly J. Phelps, Kiet Tran, Tristan Eifler, Anna I. Erickson, Andrew J. Fisher, Peter A. Beal

**Affiliations:** 1 Department of Chemistry, University of California, One Shields Avenue, Davis, CA 95616, USA; 2 Department of Molecular and Cellular Biology, University of California, One Shields Avenue, Davis, CA 95616, USA

## Abstract

Adenosine deaminases acting on RNA (ADARs) hydrolytically deaminate adenosines (A) in a wide variety of duplex RNAs and misregulation of editing is correlated with human disease. However, our understanding of reaction selectivity is limited. ADARs are modular enzymes with multiple double-stranded RNA binding domains (dsRBDs) and a catalytic domain. While dsRBD binding is understood, little is known about ADAR catalytic domain/RNA interactions. Here we use a recently discovered RNA substrate that is rapidly deaminated by the isolated human ADAR2 deaminase domain (hADAR2-D) to probe these interactions. We introduced the nucleoside analog 8-azanebularine (8-azaN) into this RNA (and derived constructs) to mechanistically trap the protein–RNA complex without catalytic turnover for EMSA and ribonuclease footprinting analyses. EMSA showed that hADAR2-D requires duplex RNA and is sensitive to 2′-deoxy substitution at nucleotides opposite the editing site, the local sequence and 8-azaN nucleotide positioning on the duplex. Ribonuclease V1 footprinting shows that hADAR2-D protects ∼23 nt on the edited strand around the editing site in an asymmetric fashion (∼18 nt on the 5′ side and ∼5 nt on the 3′ side). These studies provide a deeper understanding of the ADAR catalytic domain–RNA interaction and new tools for biophysical analysis of ADAR–RNA complexes.

## INTRODUCTION

Adenosine deaminases acting on RNA (ADARs) are editing enzymes responsible for hydrolytic deamination of adenosine (A) in the context of RNA polymers, producing inosine (I) at corresponding nucleotide positions (1–3). This reaction has a variety of functional consequences for the RNA substrates including modulating thermal stability of base-paired structures, changing the meaning of codons in mRNAs (recoding), altering splicing patterns of pre-mRNAs, changing miRNA targeting within 3′ untranslated regions (UTRs), etc. (4–10). This phenomenon is widespread in the human transcriptome with thousands of inosine sites identified (11). In addition, misregulation of A to I RNA editing is associated with human disease (12–18). Indeed, mutation of one of the human genes responsible for A to I RNA editing (*adar1*) is one cause of the inherited autoimmune disease Aicardi–Goutieres syndrome (19).

**Figure 1.**
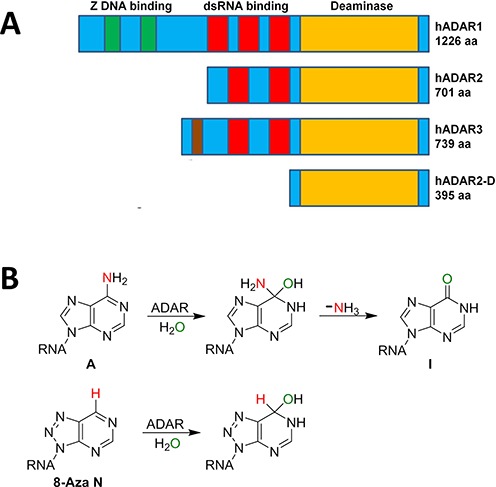
(**A**) (top): domain maps for ADAR proteins. hADAR2-D refers to the deaminase domain of human ADAR2. Yellow: deaminase (aa 299–701); red: double-stranded RNA binding (dsRBD); green: Z binding domain (ADAR1 only); brown: R-rich domain (ADAR3 only). (**B**) (top): deamination reaction catalyzed by ADARs showing high-energy intermediate with tetrahedral carbon at adenine position 6. Bottom: nucleoside analog 8-azanebularine (8-azaN) forms a mimic of deamination intermediate when hydrated (28).

Three human ADAR genes have been identified (*adars 1–3*) with ADAR1 and ADAR2 proteins having well-characterized adenosine deamination activity (2). ADAR3 is expressed in human brain but its function remains a mystery since no catalytic activity has been reported for the protein. The ADARs are modular in their structure with clearly identifiable RNA binding and deaminase domains (Figure 1A). Indeed, RNA binding is largely controlled by double-stranded RNA binding domains (dsRBDs) present in multiple copies in each ADAR protein (20). DsRBDs bind any RNA duplex greater than ∼16 bp in a sequence-independent manner (21). The presence of dsRBDs in the ADAR structure is consistent with the requirement for duplex RNA in ADAR substrates for efficient deamination to take place. However, recent studies designed to direct editing to new sites with fusion proteins containing ADAR catalytic domains suggest these domains also require duplex RNA for efficient reaction, even in the absence of dsRBDs (22–24). While the RNA-binding properties of ADARs’ dsRBDs have been well documented (25,26), few reports have focused on the RNA binding requirements of the ADAR catalytic domains.

Our laboratory recently discovered RNA substrates for human ADAR2 that are rapidly deaminated by a deletion mutant that lacks both dsRBDs (hADAR2-D) (Figure 1A) (27). Here we use one of these RNA sequences as the basis for a study of the ADAR2 catalytic domain–RNA interaction. To facilitate these experiments, we incorporated into the RNAs a nucleoside analog (8-azanebularine, 8-azaN) whose covalent hydrate is a mimic of a high-energy intermediate on the proposed deamination reaction pathway (Figure 1B) (28). The resulting high affinity of the RNA–protein complex allowed us to use electrophoretic mobility shift assays (EMSAs) and nuclease footprinting to explore catalytic domain–RNA interactions. We observed high affinity binding by hADAR2-D with short RNA duplexes (∼22 bp) derived from *Saccharomyces cerevisiae bdf2* mRNA and bearing 8-azaN at the reactive site. Formation of the stable protein–RNA complex with hADAR2-D requires duplex RNA as 8-azaN-containing single-stranded RNA does not bind. 2′-Deoxy substitution in the strand complementary to the edited strand showed a pronounced dependence on ribose sugars near the edited site suggesting catalytic domain contacts to this strand at this location. A known mutation that increases the human ADAR2 catalytic rate (E488Q) enhances binding of hADAR2-D to the 8-azaN-containing duplex RNA. Finally, trapping hADAR2-D on a 65-bp 8-azaN-containing dsRNA and subsequent cleavage with ribonuclease V1 revealed a footprint on the edited strand that extends 18 nucleotides in the 5′ direction and five nucleotides in the 3′ direction from the site of modification. These studies provide a deeper understanding of the ADAR catalytic domain–RNA interaction and new tools for biophysical analysis of ADAR–RNA complexes.

## MATERIALS AND METHODS

### General procedures

Unless otherwise stated, reagents were purchased from Fisher Scientific, Sigma-Aldrich, or Life Technologies. Brown Centrex columns were purchased from VWR. T4 polynucleotide kinase, T4 DNA ligase, molecular biology grade bovine serum albumin (BSA), chemically competent DH5α *Escherichia coli* and RNase inhibitor were purchased from New England Biolabs. γ-[^32^P] ATP was purchased from Perkin-Elmer Life Sciences. The Avian Myeloblastosis Virus (AMV) reverse transcriptase, deoxynucleotide triphosphate (dNTP) mix and RQ1 RNase free DNase were purchased from Promega. Nuclease T1 and nuclease V1 were purchased from Ambion. Pfu Ultra II was purchased from Stratagene. Dpn 1 was purchased from Invitrogen. RNA oligonucleotides were synthesized at the University of Utah DNA/Peptide Core Facility or purchased from GE Healthcare Dharmacon, Inc. or Sigma Aldrich. DNA oligonucleotides were purchased from Integrated DNA Technologies or Sigma Aldrich. Storage phosphor imaging plates from Molecular Dynamics were imaged using Molecular Dynamics 9400 Typhoon phosphorimager. Data were analyzed using Molecular Dynamics ImageQuant 5.2 software. Electrospray Ionization (ESI) mass spectrometry of oligonucleotide samples was carried out at either the Campus Mass Spectrometry Facilities, UC Davis or at Novatia, LLC. Oligonucleotide masses were determined using Mongo Oligo Mass Calculator v2.06.

### Sequences of oligonucleotides

(**a**): 5′-UUC CCC ACU UGU CAU UAG ACG UUC AGU UAG UAC CAC CAA UGA CAA UAU UGG GGA A-3′; (**b**): 5′-UUC CCC ACA UUA GAC GUU CAG UUA GUA CCA CCA AUG UGG GGA A-3′; (**b**) containing 8-azaN: 5′-UUC CCC ACA UUX GAC GUU CAG UUA GUA CCA CCA AUG UGG GGA A-3′ where X is 8-azaN; (**c**): 5′-CCC CAC AUU AGA CGU UCA GUU AGU ACC ACC AAU GUG GGG-3′; (**c**) containing 8-azaN: 5′-CCC CAC AUU XGA CGU UCA GUU AGU ACC ACC AAU GUG GGG-3′; 22-nt native top strand: 5′-UUC CCC ACA UUA GAC GUU CAG U-3′; 22-nt 8-azaN containing top strand: 5′-UUC CCC ACA UUX GAC GUU CAG U-3′; bottom strand of (**d**): 5′-ACU GAA CCA CCA AUG UGG GGA A-3′; (**e**) top strand: 5′-CAU UAX GGU GGG UGG AAU AGU AUA ACA-3′; (**e**) bottom strand: 5′-UGU UAU AGU AUC CCA CCU ACC CUG AUG-3′; (**f**) bottom strand: 5′-ACU GAA CCA CCA AUG tgg gga a-3′ where lower case letters are deoxynucleotides; (**g**) bottom strand: 5′-act gaa cCA CCA AUG UGG GGA A-3′; (**h**) bottom strand: 5′-ACU GAA Cca cca atg UGG GGA A-3′; (**i**) bottom strand: 5′-ACU GAA CGA CCA AUG UGG GGA A-3′; (**j**) bottom strand: 5′-ACU GAA CGU CCA AUG UGG GGA A-3′; 65-nt bottom strand: 5′-CAA ACA CCA UUG UCA CAC UCC AAC UGA ACG ACC AAU GUG GGG AAA UAG GAU UCA UAU UAG GAG AU-3′; 65-nt left sequence: 5′-AUC UCC UAA UAU GAA UCC UAU-3′; 65-nt right sequence: 5′-UGG AGU GUG ACA AUG GUG UUU G-3′; primer for (**a**): 5′-TTG TCA TTG GTG GTA CTA ACT GAA CGT-3′; primer for (**b**): 5′-CCC ACA TTG GTG GTA CTA ACT GAA CGT-3′; 65-nt DNA splint: 5′-CAA ACA CCA TTG TCA CAC TCC AAC TGA ACG TCT AAT GTG GGG AAA TAG GAT TCA TAT TAG GAG AT-3′.

### Oligonucleotide synthesis and purification

The 8-azaN phosphoramidite was synthesized as previously described (28) and RNAs were synthesized as previously described (29). Single-stranded RNAs (**a**) and (**b**) and DNA oligonucleotides were purified by denaturing polyacrylamide gel electrophoresis and visualized using UV shadowing. Bands were excised from the gel, crushed and soaked overnight at 4°C in 0.5-M NH_4_OAc, 0.1% sodium dodecyl sulphate (SDS) and 0.1-mM ethylenediaminetetraacetic acid (EDTA). Polyacrylamide fragments were removed using a 0.2-μm filter (Centrex filter or Spin-X column) followed by phenol-chloroform extraction and ethanol precipitation. The RNA solutions were lyophilized to dryness, re-suspended in nuclease-free water, quantified by absorbance at 260 nm and stored at −70°C. The RNAs were later heated at 95°C for 5 min and then slowly cooled to room temperature in 10-mM Tris-HCl, 0.1-mM EDTA pH 7.5, 100-mM NaCl to allow them to refold. All other RNA oligonucleotides were purified in the same manner except for the following modifications: samples were crushed and soaked in 0.5-M NH_4_OAc and 0.1-mM EDTA and were desalted by either ethanol precipitation and a 70% ethanol wash or Sep-Pak instead of phenol-chloroform extraction and ethanol precipitation. ESI-mass spectrometry (MS) values for RNAs are as follows: (**b**) containing 8-azaN: calcd. 13698.9, obsd. 13700.5; (**c**) containing 8-azaN: calcd. 12434.5, obsd. 12434.6; 22-nt 8-azaN containing top strand: calcd. 6881.0, obsd. 6881.9.

### Protein overexpression and purification

The hADAR2-D E488Q mutant was prepared using polymerase chain reaction mutagenesis with template previously described (30) and the following mutagenic primers with mutation position underlined: 5′-CAA AAT AGA GTC TGG TCA GGG GAC GAT TCC AG-3′ and 5′-CTG GAA TCG TCC CCT GAC CAG ACT CTA TTT TG-3′. N-terminal histidine-tagged human ADAR2 deaminase domain (aa 299–701) (hADAR2-D) and hADAR2-D E488Q mutant proteins were expressed in *S. cerevisiae* strain BCY123 and purified as described previously (30) omitting the Tobacco Etch Virus (TEV) cleavage, heparin and gel filtration columns. Proteins concentrations were determined using BSA standards visualized by SYPRO Orange staining of SDS-polyacrylamide gels and the purified proteins were stored in 20-mM Tris-HCl, pH 8.0, 100-mM NaCl, 20% glycerol, 1-mM 2-mercaptoethanol at −70°C.

### ^32^P labeling of oligonucleotides and duplex formation

DNA primers for primer extension assays with (**a**) and (**b**) were radiolabeled with [γ-^32^P] at the 5′ end as described previously (31). Single-stranded RNAs (**b**), (**b**) containing 8-azanebularine and (**e**) top strand were labeled as previously described in (32) and redissolved in 10-mM Tris-HCl, 0.1-mM EDTA pH 7.5 and 100-mM NaCl to a final concentration no greater than 45 nM. Labeled RNAs were refolded by heating for 5 min at 95°C and then allowing them to slowly cool to room temperature. The 22-nt native top strand and 22-nt 8-azanebularine containing top strand were labeled according to NEB's Radioactive Labeling with T4 PNK using half the recommended volume. Samples were purified as previously described in (32) and RNAs were redissolved in 10-mM Tris-HCl, 0.1-mM EDTA pH 7.5 and 100-mM NaCl to a final concentration no greater than 45 nM. For duplex formation, the complementary strand was added in 10-fold excess. The samples were then heated for 5 min at 95°C and allowed to slowly cool to room temperature.

### ADAR deamination kinetics

Deamination reactions with RNAs (**a**) and (**b**) were carried out under the conditions previously described and products ratios were determined using a primer extension assay (27). Deaminated RNAs were re-suspended in 1-μl 5X AMV reverse transcription buffer and 4-μl-radiolabeled DNA primer (∼4000 cpm per sample). Samples were incubated at 62°C for 15 min and then cooled on ice for 5 min. After cooling, a mixture containing 1-μl 5X AMV reverse transcription buffer, 0.5-μl AMV reverse transcriptase, 1.5-μl nuclease-free water and 2-μl dNTP mix (50-μM 2′-Deoxycytidine-5′-Triphosphate (dCTP), 2′-Deoxyadenosine-5′-Triphosphate (dATP), 2′-Deoxyguanosine-5′-Triphosphate (dGTP) and 2′,3′-Dideoxythymidine-5′-Triphosphate (ddTTP)) was added to each sample. Samples were then incubated at 42°C for 45 min. Reverse transcription reactions were quenched with 7-μl 80% formamide, 10-mM EDTA pH 8.0. Samples were denatured by heating at 95°C for 3 min then loaded onto a 12% denaturing polyacrylamide gel. The gel was visualized by overnight exposure to storage phosphor imaging plates and quantified by volume integration using ImageQuant software. Data were fitted to the equation *y* = *A*(1-*e*^(-kobs*t)^), where *A* is the fitted reaction end point, *k*_obs_ is the fitted rate constant (min^−1^) and *y* is the fraction product at time *t*. Graphs were generated using KaleidaGraph (Synergy Software).

### EMSAs with hADAR2-D

Samples containing 2.5-nM RNA and different concentrations of hADAR2-D (3200, 1000, 750, 500, 250, 100, 75, 50, 25, 10, 5 and 0 nM) were equilibrated in 20-mM Tris-HCl, pH 7, 6% glycerol, 0.5-mM DTT, 60-mM KCl, 20-mM NaCl, 0.1-mM 2-mercaptoethanol, 1.5-mM EDTA, 0.003% NP-40, 160-units/ml RNasin, 100-μg/ml BSA and 1.0-μg/ml yeast tRNA for 30 min at 30°C. Samples were loaded on a 6% 80:1 native polyacrylamide gel, dried at 80°C for 1 h and imaged. Bands were quantified by volume integration using ImageQuant software such that all radioactive signal not found in the free RNA band was quantified as bound RNA. The data were fitted to the following equation: Fraction bound = *A* * [protein]/([protein] + *K*_d_), where the *K*_d_ is the fitted dissociation constant and *A* is the fitted maximum fraction of RNA bound.

### Thermal denaturation studies

RNA duplexes (**d**), (**f**), (**g**) and (**h**) were hybridized at a concentration of 0.5 μM in 10-mM Tris-HCl, 0.1-mM EDTA pH 7.5 and 100-mM NaCl by heating at 95°C for 5 min and allowing them to slowly cool to room temperature. The melting temperature (*T*_M_) was determined for each as previously described (33).

### Preparation of 65-bp duplex containing 8-azaN

The 65-nt RNA strand containing 8-azaN used in RNase V1 footprinting experiments was prepared using a three-piece-splinted ligation (34,35). The three RNAs ligated together to form this strand are described here as: 65 nt left, 22-nt 8-azaN top strand and 65 nt right (see Supplementary Figure S1 for ligation strategy). The 65-nt left sequence was ^32^P labeled using NEB's Radioactive Labeling with T4 PNK protocol with half the recommended volume. The 22-nt 8-azaN top strand and 65-nt right sequences were phosphorylated by incubating 20 pmols of RNA with 1X T4 DNA Ligase Buffer and 10 units of PNK for 1 h at 37°C. The phosphorylated 65-nt right sequence, the phosphorylated 22-nt 8-azaN top strand and the labeled 65-nt left sequence were mixed together in equal amounts with a 65-nt DNA splint in 1X T4 DNA Ligase buffer such that the final concentration was 170 nM for the three RNAs and 150 nM for the DNA splint. The mixture was then heated to 95°C for 5 min and allowed to slowly cool to room temperature. T4 DNA ligase (14 000 U) was added and the reaction was allowed to proceed overnight at 16°C. The reaction was then phenol-chloroform extracted, ethanol precipitated, 70% ethanol washed and dried. The sample was re-dissolved in 1X RQ1 Reaction Buffer and treated with 50 U of RQ1 RNase-Free DNase for 1 h at 37°C, loaded onto a 12% denaturing gel polyacrylamide gel and visualized using phosphor imaging. RNA bands were excised from the gel, crushed and soaked overnight at 4°C in 0.5-M NH_4_OAc, 0.1% SDS and 0.1-mM EDTA. Samples were filtered through a Spin-X column, phenol-chloroform extracted, ethanol precipitated, 70% ethanol washed and dried. The ligated 65-nt RNA was redissolved in 10-mM Tris-HCl, 0.1-mM EDTA pH 7.5 and 100-mM NaCl and mixed with the 65-nt bottom strand to a final concentration no greater than 20 nM for the ligated strand and 200 nM for the complementary strand.

### Nuclease V1 footprinting

Samples containing no greater than 3-nM RNA with various concentrations of hADAR2-D (0, 10, 15, 20, 25, 75, 100, 150 and 200 nM) were equilibrated in 10-mM Tris-HCl, pH 7.1, 0.2% glycerol, 60-mM KCl, 25-mM NaCl, 10-μM 2-mercaptoethanol, 15-μM EDTA, 100-μg/ml BSA, 0.3-mM MgCl_2_ and 1.0-μg/ml yeast tRNA for 30 min at 30°C. Samples were then incubated with 0.0007 units of RNase V1 for 7.5 min. RNase V1 Inactivation/Precipitation Buffer was added to each sample followed by ethanol precipitation. Nuclease T1 digestion was also carried out on the single-stranded-labeled 65-nt RNA using the RNA Structure-Function Protocol from Life Technologies with 0.7 units for 15 min. Samples were then treated with 20 units of PNK in 1X PNK buffer for 30 min at 37°C. Samples were heated at 98°C for 10 min in denaturing loading buffer and fractionated on a 12% denaturing gel, dried at 80°C for 1 h and imaged.

## RESULTS

### A short RNA substrate for hADAR2-D

**Figure 2.**
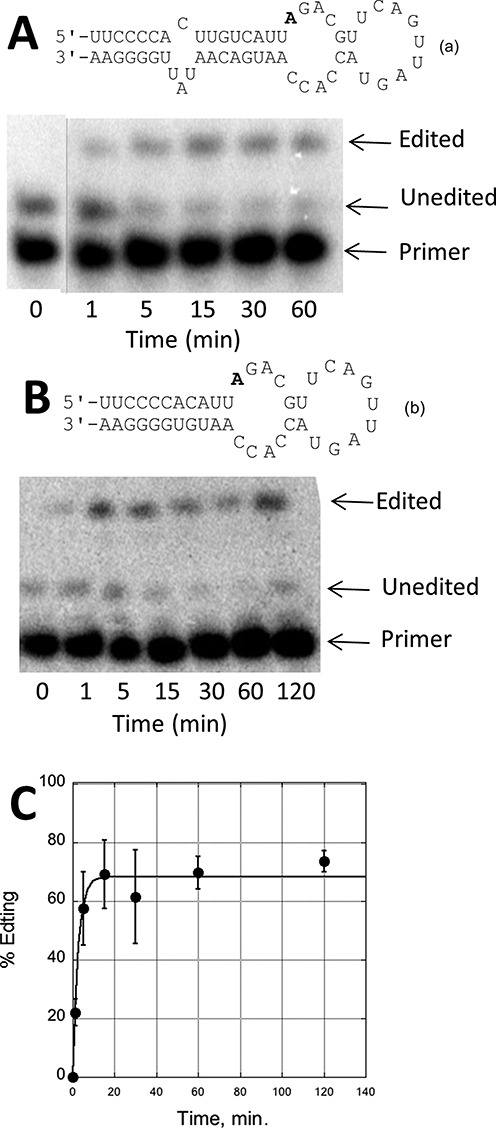
(**A**) (top): predicted secondary structure for 55 nucleotides flanking a previously identified ADAR2 site in the *S. cerevisiae* bdf2 mRNA shown bolded (**a**) (27). Bottom: primer extension analysis for hADAR2-D deamination products with (**a**). Line indicates removal of unrelated lanes. (**B**) (top): secondary structure for 43-nt bdf2-derived substrate (**b**) with reactive adenosine shown bolded. Bottom: primer extension analysis for hADAR2-D deamination products with (**b**). (**C**) Plot of deamination product as a function of time for (**b**) and hADAR2-D.

In a previous report, we described RNAs that react efficiently with hADAR2-D, a deletion mutant of hADAR2 that lacks both dsRBDs (27). Importantly, no other adenosines near this editing site were deaminated with high efficiency by hADAR2-D demonstrating the selectivity of the reaction (Supplementary Figure S2). It occurred to us that these RNAs would be useful in defining catalytic domain–RNA interactions, particularly if they could be shortened to a length accessible by chemical synthesis and retain high hADAR2-D reactivity. Shorter RNA substrates would allow for routine incorporation of nucleoside analogs known to facilitate studies of the ADAR mechanism, such as 2-aminopurine ribonucleoside (36) and 8-azanebularine (28,37,38). Here we tested the 55 nt RNA (**a**) containing the reactive site previously identified in the *S. cerevisiae* bdf2 mRNA as a substrate for hADAR2-D (Figure 2) (27). The reaction proceeded rapidly (*k*_obs_ > 2 min^−1^) under our standard assay conditions (10-nM RNA, 150-nM hADAR2-D, 15-mM Tris-HCl, pH 7.1, 3% glycerol, 0.5-mM DTT, 60-mM KCl, 1.5-mM EDTA, 0.003% NP-40, 160-units/ml RNasin and 1.0-μg/ml yeast tRNA^Phe^ at 30°C). Reducing the size of the stem by four base pairs and removing the 1 × 3 asymmetrical internal loop led to RNA (**b**) (Figure 2). This RNA was also shown to be a good substrate for hADAR2-D albeit with a reduced rate (*k*_obs_ = 0.38 ± 0.06 min^−1^) compared to that observed for (**a**) under the same conditions. Nevertheless, this reaction rate is comparable (or superior) to those observed for other model substrates used to study the reaction of full length ADARs (e.g. GluR B R/G site (29,38–40), GluR B Q/R site (26), NEIL1 K/R site (35)).

### hADAR2-D binds tightly to a 43-nt hairpin stem RNA bearing 8-azaN

**Figure 3.**
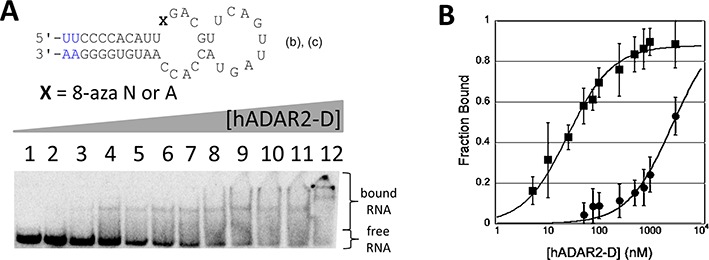
(**A**) (top): secondary structure for (**b**) and (**c**) with X = A or 8-azaN at reactive site. The 39-nt RNA (**c**) lacks the four nucleotides shown in blue in figure. Bottom: autoradiogram of gel used to resolve bound from free RNA in EMSA with hADAR2-D and (**b**) where X = 8-azaN. Lanes 1–12: 0, 5, 10, 25, 50, 75, 100, 250, 500, 750, 1000 and 3200-nM ADAR2 added. (**B**) Plot of fraction (**b**) bound as a function of hADAR2-D concentration: squares: X = 8-azaN; circles: X = A.

8-AzaN is an adenosine analog useful for the study of the ADAR reaction since it traps the ADAR protein bound at the reactive site in the RNA (Figure 1B) (28). The structural changes to adenosine (8-aza, 6-desamino) facilitate covalent hydration but prevent breakdown of that hydrate to deaminated product (28). Since the 8-azaN hydrate resembles a high-energy intermediate on the reaction pathway, one that is likely stabilized by the enzyme, high affinity binding to the ADAR protein is realized (28). However, tight binding is only observed if the site of 8-azaN modification is a rapidly deaminated adenosine in the native RNA (28,38). Prior to this work, no synthetically accessible RNA had been reported that was deaminated by hADAR2-D with a rate high enough to suggest 8-azaN substitution would lead to tight binding (i.e. at least one dsRBD was required for a rapid reaction). We wished to trap hADAR2-D bound to 8-azaN-modified RNAs to study catalytic domain–RNA interactions and to remove the complicating effects of the promiscuous binding dsRBDs. Thus, we used the previously reported 8-azaN phosphoramidite to prepare a 43-nt RNA (**b**) with the analog at the reactive site (Figure 3). Using EMSA, we showed that hADAR2-D binds tightly to 8-azaN-modified (**b**) (*K*_d_ = 27 ± 16 nM), whereas (**b**) with A at the reactive site binds over 100-fold weaker (*K*_d_ > 3200 nM) (Figure 3). Interestingly, 39-nt RNA (**c**) lacking two base pairs on the end of the stem binds hADAR2-D less tightly that (**b**) (*K*_d_ = 310 ± 60) suggesting the catalytic domain requires the longer duplex (Figure 3 and Supplementary Figure S3).

### An intermolecular duplex bearing 8-azaN binds tightly to hADAR2-D

**Figure 4.**
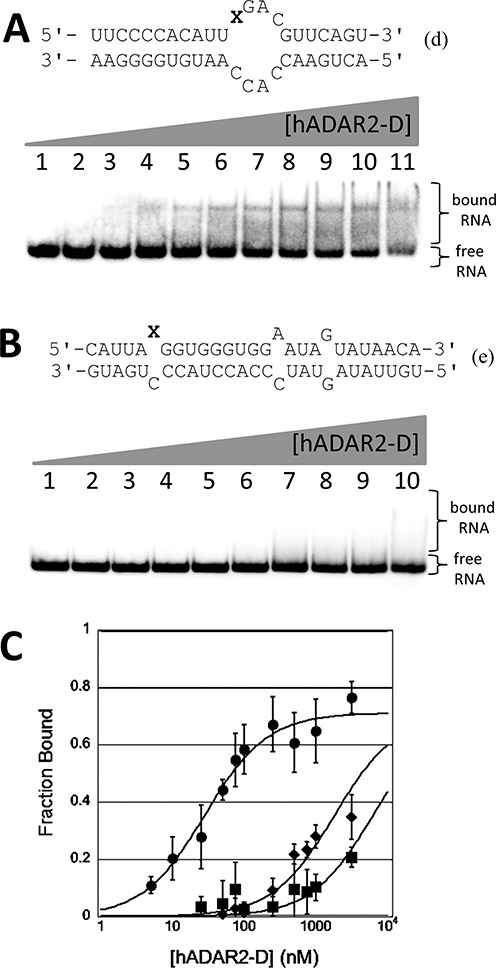
(**A**) (top): predicted secondary structure for bdf2-derived RNA (**d**). Bottom: autoradiogram of gel used to resolve bound from free RNA in EMSA with hADAR2-D and (**d**) where X = 8-azaN. Lanes 1–11: 0, 5, 10, 25, 50, 75, 100, 250, 500, 1000 and 3200-nM ADAR2 added. (**B**) (top): predicted secondary structure for GluR B R/G site RNA (**e**). Bottom: autoradiogram of gel used to resolve bound from free RNA in EMSA with hADAR2-D and (**e**) where X = 8-azaN. Lanes 1–10: 0, 25, 50, 75, 100, 250, 500, 750, 1000 and 3200-nM ADAR2 added. (**C**) Plot of fraction RNA bound as a function of hADAR2-D concentration: circles: (**d**) where X = 8-azaN; diamonds: (**e**) where X = 8-azaN; squares: (**d**) where X = A.

While the 43-nt RNA (**b**) is accessible by chemical synthesis, scale-up of the synthesis of this RNA proved challenging. We then asked whether an intermolecular duplex that retains the sequence and secondary structural elements found near the editing site in (**b**) would also bind tightly to hADAR2-D. Thus, intermolecular duplex RNA (**d**), with the hairpin loop of (**b**) replaced with additional base paired structure, was prepared bearing either A or 8-azaN at the reactive site (Figure 4A). When modified with 8-azaN, this RNA binds hADAR2-D with a *K*_d_ = 30 ± 8 nM, very similar to the affinity measured to hairpin–stem structure (**b**) (*K*_d_ = 27 ± 16 nM) (Figure 4). As observed with (**b**), when A is placed at the reactive site, the hADAR2-D affinity measured by quantitative EMSA is substantially lower (*K*_d_ > 3200 nM) (Figure 4). Importantly, the formation of a stable hADAR2–D/RNA complex is dependent on the sequence context of the 8-azaN and its positioning within the duplex. A model substrate for the GluR B R/G editing site has been used to study the reaction of full length ADAR2 and a deletion mutant lacking one of the dsRBDs (28,37,40,41). However, we find that, hADAR2-D binds poorly to this RNA when 8-azaN is positioned at the reactive site (Figure 4).

### 8-AzaN-containing duplexes used to define important structural features for ADAR2 catalytic domain binding

**Figure 5.**
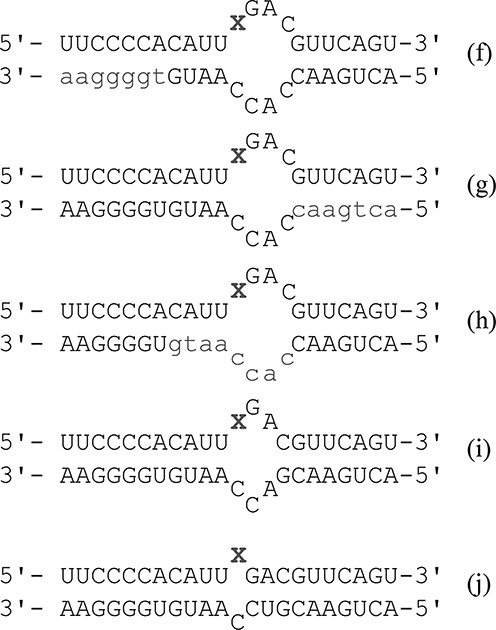
Duplexes f–j with varying complementary strand structures. Lower case gray indicates a 2′-deoxynucleotide. X = 8-azaN.

Since a high affinity complex could be observed by EMSA with (**d**) bearing 8-azaN at the reactive site and hADAR2-D, we evaluated the effects of changing the sequence and structure of the complementary strand. This was carried out with duplexes (**f–j**) (Figure 5) that introduce blocks of 2′-deoxynucleotides near the 3′ end of the complementary strand (**f**), near the 5′ end (**g**) and middle (**h**). In addition, sequence changes were made to pair mismatched bases in the internal loop (duplexes (**i**) and (**j**)). EMSA was then performed with hADAR2-D and each of these duplexes along with the 8-azaN-containing single strand and a duplex with a fully 2′-deoxy complementary strand as controls. No stable complex was observed with either the 8-azaN containing single strand or the duplex with an entirely DNA complement (Supplementary Figure S4) further establishing the strict requirement for duplex RNA in the substrate for ADAR2's catalytic domain. Duplexes with DNA/RNA chimeric complementary strands (**f, g** and **h**) form complexes of varying stability (Table 1 and Supplementary Figure S5). All three complexes are less stable than observed with (**d**). However, (**h**) with the 2′-deoxy block in the middle of the complementary strand binds particularly poorly with a measured *K*_d_ = 342 ± 88 nM. Thermal denaturation (*T*_M_) studies were carried out with these three duplexes along with parent duplex (**d**) to allow a comparison of the effects of these changes on duplex stability and hADAR2-D binding (Table 2). Importantly, (**d**) and (**h**) have the same thermal stability but bind hADAR2-D with >10-fold difference in dissociation constant. Thus the ribose requirement is not simply due to an effect on duplex stability and is more likely a result of interactions between the catalytic domain and this strand that require ribose(s). Sequence changes that eliminate mismatches adjacent to the editing site (RNAs (**i**) and (**j**)) improve binding to hADAR2-D. This provides additional evidence for the importance of duplex RNA structure surrounding the editing site for ADAR2 catalytic domain interactions.

**Table 1.** Dissociation constants for hADAR2-D binding to 8-azaN RNAs

RNA	*K*_d_ (nM)^a^
(b)	27 ± 16
(c)	310 ± 60
(d)	30 ± 8
(e)	>3200
(f)	114 ± 11
(g)	99 ± 37
(h)	342 ± 88
(i)	15 ± 2
(j)	10 ± 2

^a^Samples were equilibrated in 20-mM Tris-HCL, pH 7.1, 6% glycerol, 0.5-mM DTT, 60-mM KCl, 20-mM NaCl, 0.1-mM 2-mercaptoethanol, 1.5-mM EDTA, 0.003% NP-40, 160-units/ml RNasin, 100-μg/ml BSA and 1.0-μg/ml yeast tRNA for 30 min at 30°C.

**Table 2.** Thermal melting temperature (*T*_M_) for duplex RNAs

RNA^a^	*T*_M_^b^
(d)	53 ± 1
(f)	44 ± 0.4
(g)	49 ± 0.3
(h)	53 ± 0.3

^a^*T*_M_ values were measured for editing substrate RNAs (where X = 8-azaN in Figure 5).

^b^Value reported is in °C and is the average of three independent measurements ± standard deviation.

**Figure 6.**
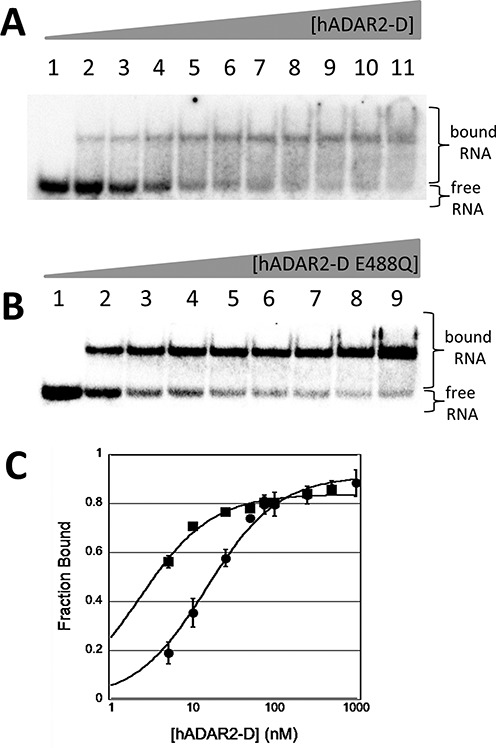
(**A**) Autoradiogram of gel used to resolve bound from free RNA in EMSA with hADAR2-D and (i) where X = 8-azaN. Lanes 1–11: 0, 5, 10, 25, 50, 75, 100, 250, 500, 1000 and 3200-nM ADAR2 added. (**B**) Autoradiogram of gel used to resolve bound from free RNA in EMSA with hADAR2-D E488Q and (i) where X = 8-azaN. Lanes 1–9: 0, 5, 10, 25, 50, 75, 100, 250 and 500-nM ADAR2 added. (**C**) Plot of fraction (i) bound as a function of protein concentration: squares: hADAR2-D E488Q; circles: hADAR2-D.

Recently, Kuttan and Bass identified a mutant of human ADAR2 (E488Q) with enhanced catalytic activity (i.e. higher single turnover rate constant for deamination of model substrates compared to the wild-type enzyme)(40). Given that binding affinity for 8-azaN-modified RNAs correlates with catalytic rates (28,38), we predicted this mutation in hADAR2-D would further enhance binding affinity to the 8-azaN-modified RNAs described here. To test this, we used EMSA to measure the binding affinity of the hADAR2-D E488Q mutant for RNA (**i**) (Figure 6). As predicted, this mutant protein bound more tightly than wild type under our assay conditions (hADAR2 E488Q *K*_d_ < 5 nM, hADAR2-D *K*_d_ = 15 ± 2 nM). Interestingly, the hADAR2-D E488Q mutant showed substantially less smearing in the gel than did the wild-type hADAR2-D suggesting differences in dissociation rates for the two complexes (42). Furthermore, the hADAR2-D E488Q mutant had weak binding to the dsRNA lacking 8-azaN, similar to that observed for the wild-type hADAR2-D (Supplementary Figure S6).

### Nuclease footprinting of hADAR2-D trapped on a long 8-AzaN-containing duplex

**Figure 7.**
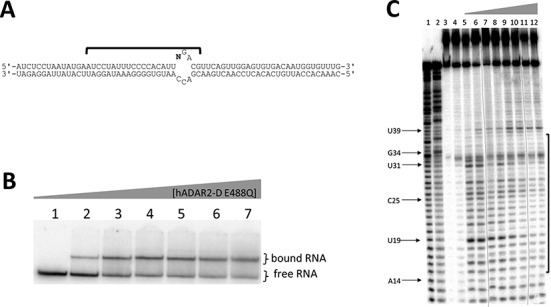
(**A**) Secondary structure of the 65-bp 8-azaN-containing structure with *N* = 8-azaN at reaction site. (**B**) Autoradiogram of gel used to resolve bound from free RNA in EMSA with hADAR2-D E488Q and the long 8-azaN-containing structure. Samples were equilibrated in 10-mM Tris-HCl, pH 7, 0.2% glycerol, 60-mM KCl, 25-mM NaCl, 10 μM 2-mercaptoethanol, 15-μM EDTA, 100 μg/ml BSA, 0.3-mM MgCl_2_ and 1.0-μg/ml yeast tRNA for 30 min at 30°C. Lanes 1–7: 0, 25, 50, 75, 100, 150 and 200-nM ADAR2 added. (**C**) Nuclease V1 footprint of the long 8-azaN-containing RNA. Phosphor autoradiogram of a 12% denaturing polyacrylamide gel separating the 5′-end-labeled RNA cleavage products, nucleotides protected from V1 cleavage are identified with a bracket. Reaction conditions as labeled: lane 1, T1; lane 2, T1 dephosphorylated; lane 3, untreated RNA; lane 4, no nuclease V1; lanes 5–12: 0, 10, 20, 25, 50, 75, 150 and 200-nM ADAR2 added. Each line indicates the removal of a lane containing an over-digested sample.

Nuclease footprinting experiments are valuable for defining binding characteristics of nucleic acid-binding proteins (43). However, prior footprinting experiments with ADARs have been carried out with proteins that contain dsRBDs and, as such, have been complicated by dsRBD binding to the RNA (44). Bass *et al.* observed a faint 16-nt protection, with 8-nt protection on the 5′ side and 7-nt protection on the 3′ side at low concentrations of ADAR2, but results were obscured at higher concentrations because the entire RNA stem loop used in the study was protected from cleavage. This also correlated to a second shift in EMSA, leading to the conclusion that the entire RNA is coated with ADAR2 at the higher concentrations. We reasoned that if the bdf2-derived sequence bearing 8-azaN at the editing site were present in a longer otherwise unmodified RNA duplex, differential reactivity to a duplex RNA-specific nuclease should be observed in the presence of hADAR2-D and new information about ADAR catalytic domain binding would be obtained. To generate such a structure, we used a splinted ligation strategy to insert RNA (**i**) into a long (65 bp) duplex (Figure 7A). Importantly, EMSA with this RNA and hADAR2-D E488Q showed formation of a single protein–RNA complex (Figure 7B). No higher order complexes are observed under these gel shift conditions typical of ADAR constructs bearing dsRBDs binding long duplex RNAs.

Ribonuclease V1 is a duplex-RNA specific nuclease useful for footprinting dsRNA-binding proteins (45–48). In the absence of hADAR2-D, V1 cleaves the 65-bp duplex at multiple nucleotides flanking the 8-azaN modification site (Figure 7C, lane 5). In the presence of hADAR2-D E488Q, the V1 reactivity pattern changes significantly with some positions protected while some nucleotides become hyperreactive to V1 (Figure 7C, lanes 9–12). Strong protection from V1 cleavage is observed at nucleotides U19, C25 and U31 in the presence hADAR2-D. In addition, reactivity at U39 is enhanced in the hADAR2-D complex. In all, hADAR2-D protects a region of ∼23 nt on the edited strand from V1 cleavage. This footprint is asymmetrically disposed around the 8-azaN modification with more RNA protected on the 5′ side (∼18 nt) of the 8-azaN modification than on the 3′ side (∼5 nt).

## DISCUSSION

Small RNAs that are efficiently processed by modifying enzymes are useful for biophysical and mechanistic studies of the enzyme–RNA interaction. One advantage lies in the ability to incorporate nucleoside analogs at specific positions in the RNA. In the case of the ADARs, incorporation of 2-aminopurine into model substrates allows one to study the base-flipping step of the reaction by monitoring protein-induced fluorescence changes (36,40,49). Incorporation of 8-azaN enables mechanism-based trapping of the ADAR–RNA complex (28). Earlier work, however, used RNAs that were poor substrates for the catalytic domain of hADAR2 and were, thus, inappropriate for the study of this isolated domain. However, the sequence and structure of the bdf2-derived RNAs used here lead to efficient catalysis by hADAR2-D and the higher reaction rates observed translate into tight binding to hADAR2-D when 8-azaN is present in the RNA constructs. This allowed us to carryout EMSA and footprinting experiments to study the interaction between the hADAR2 catalytic domain and substrate RNAs without catalytic turnover complicating the analysis.

Importantly, no high affinity hADAR2-D complex was observed with the GluR B R/G site model RNA bearing 8-azaN (Figure 4) (28). It appears that productive interactions with the ADAR2 catalytic domain are dependent on the local sequence and/or positioning on the duplex of the reactive nucleotide. Considering differences between bdf2-derived RNA (**i**) that binds tightly to hADAR2-D and the GluR B R/G RNA (**e**) that does not, one is tempted to conclude that a duplex longer than five base pairs on the 5′ side of the editing site is required for full binding to the catalytic domain. This idea is consistent with the observation that the 8-azaN-containing 39 mer (**c**) binds more weakly than does 43 mer (**b**) that has two more base pairs 5′ to the editing site (Figure 3). In addition, the hADAR2-D footprint on the 65-bp duplex showed protection from V1 cleavage 18 nt 5′ of the 8-azaN modification (Figure 7). Analysis of the crystal structure of hADAR2-D indicates the distance from the zinc site to the protein's edge on the likely RNA binding surface is ∼30 Å, corresponding to roughly 13 bp of A-form helix (Supplementary Figure S7). This suggests hADAR2-D prevents V1 access to the RNA beyond the furthest contact point in the 5′ direction from the editing site, possibly due to the steric demand of the nuclease in approaching its substrate. However, additional structural studies with the protein–RNA complex are required to determine the specific hADAR2-D residues making contacts to the RNA. Given the rather long duplex protected in the 5′ direction from the editing site, it appears that full binding to the catalytic domain is not possible when editing occurs on substrates like the GluR B R/G site RNA since the duplex structure on the 5′ side of the editing site is too short. In cases like this, binding elsewhere on the RNA by at least one dsRBD is required for an efficient ADAR reaction (26,50). The dsRBD binding compensates for the suboptimal catalytic domain binding. Indeed, binding sites for hADAR2's dsRBDs are present on the GluR B R/G site duplex 3′ to the editing site (49,51). Furthermore, nuclear magnetic resonance data show hADAR2's dsRBDII can directly contact the guanosine immediately 3′ of the edited A in this RNA (25,51). Together these studies suggest that full length hADAR2 edits the GluR B R/G site in a complex with the dsRBDs bound to the duplex 3′ to the editing site and the catalytic domain partially bound to the RNA with contacts flanking the edited A. However, it is possible that, as the reaction proceeds, dsRBDs disengage from initial binding sites to allow the catalytic domain to interact with multiple nucleotides surrounding the editing site.

Interestingly, the modular nature of the ADAR genes has led to the suggestion that ADARs acquired dsRBDs late in evolution (52). Our results suggest that the acquisition of dsRBDs would have allowed ADARs to process more sites since the catalytic domain's requirement for duplex structure 5′ to the editing site would have been relaxed. Furthermore, the high affinity observed for hADAR2-D binding to RNAs (**i**) and (**j**) suggests an ancestral ADAR target may have been similar in structure to these (52).

hADAR2-D is clearly duplex RNA-dependent as indicated from our studies of the effects of changes to the editing site complementary strand (Figure 5 and Tables 1 and 2). High affinity binding requires both strands and is particularly sensitive to the presence of 2′-hydroxyls on the strand opposite and proximal to the editing site. These observations are consistent with recently published results from Stafforst *et al.* who showed that an editing site complementary strand could be extensively modified with 2′-O-methyl groups at nucleotides distal to the editing site but not at proximal nucleotides (22).

The insight and new RNAs reported here will continue to be useful for defining characteristics of the interaction between hADAR2's catalytic domain and RNA. For instance, 2-aminopurine-modified catalytic domain substrates can now be used to define the role of ADAR domains in the base-flipping step of the reaction (31,40,49). In addition, efforts to crystallize an ADAR–RNA complex should benefit from this work. The hADAR2-D protein has been previously crystallized in the absence of RNA (53) and now high affinity RNA ligands are known for this protein. Furthermore, our footprinting results provide guidance to the design of 8-azaN-containing RNAs for crystallization trials.

## SUPPLEMENTARY DATA

Supplementary Data are available at NAR Online.

SUPPLEMENTARY DATA
