# Supplementary material for: Recognition of duplex RNA by the deaminase domain of the RNA editing enzyme ADAR2
Source: Nucleic Acids Res. 2015 Jan 6;43(2):1123–32. doi: 10.1093/nar/gku1345 (PMC4333395; doi:10.1093/nar/gku1345)
Supplement: SUPPLEMENTARY DATA [file supp_43_2_1123__index.html]

Recognition of duplex RNA by the deaminase domain of the RNA editing enzyme ADAR2 — SUPPLEMENTARY DATA 

# Recognition of duplex RNA by the deaminase domain of the RNA editing enzyme ADAR2

## SUPPLEMENTARY DATA

**Files in this Data Supplement:**

- SUPPLEMENTARY DATA
