# Supplementary material for: Recognition of duplex RNA by the deaminase domain of the RNA editing enzyme ADAR2
Source: Nucleic Acids Res. 2015 Jan 6;43(2):1123–32. doi: 10.1093/nar/gku1345 (PMC4333395; doi:10.1093/nar/gku1345)
Supplement: SUPPLEMENTARY DATA [file supp_gku1345_nar-02963-f-2014-File009.pdf]

Supporting information:

Recognition of Duplex RNA by the Deaminase Domain of the RNA editing Enzyme ADAR2

Kelly J. Phelps[1], Kiet Tran[1], Tristan Eifler[1], Anna I. Erickson[2], Andrew J. Fisher[1,2] and Peter A. Beal[1]*

[1]Department of Chemistry and [2]Department of Molecular and Cellular Biology, University of California, One Shields Ave, Davis, CA 95616, USA

Contents of Supporting Information:

Supplementary Figures

Supplementary Figure 1…...S2

Supplementary Figure 2….S2

Supplementary Figure 3….S3

Supplementary Figure 4….S3

Supplementary Figure 5….S4

Supplementary Figure 6….S4

Supplementary Figure 7….S5

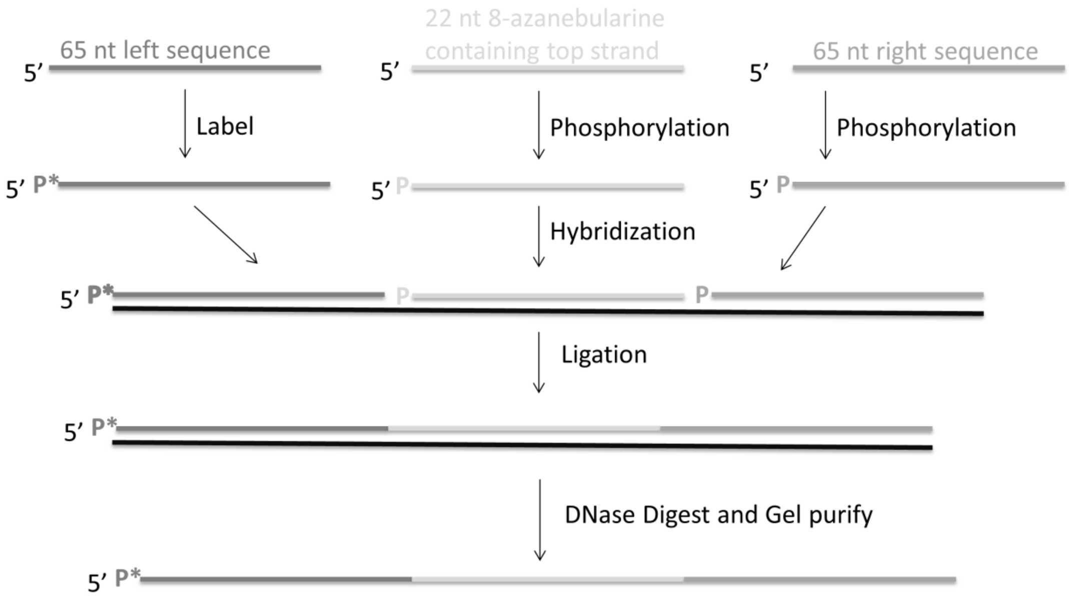

Supplementary Figure 1: Ligation strategy to prepare 8-aza-N containing 65 nt RNA. The long 8-azaN-containing structure was created by ligating three strands of RNA. First, the 22 nt 8-azaN containing top strand and 65 nt right sequence were phosphorylated and the 65 nt left sequence was ^{32}P labeled. The three oligonucleotides were hybridized to a DNA splint and ligated. The DNA splint was then removed by DNase treatment and the ligated long 8-azaN-containing RNA was gel purified.

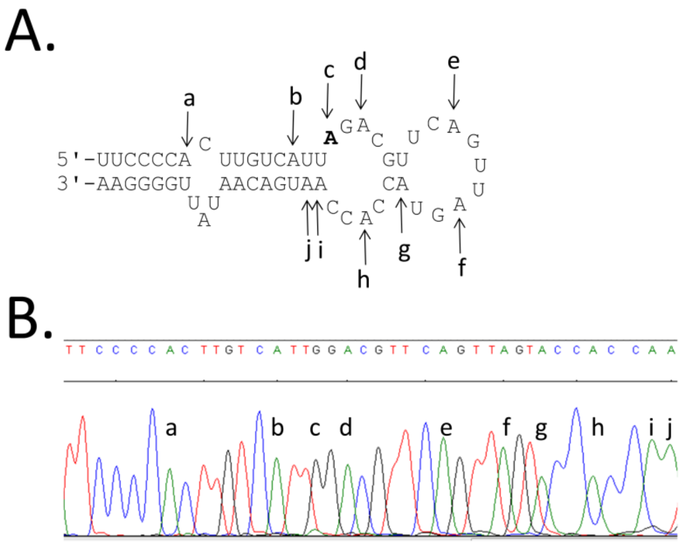

Supplementary Figure 2: (A) Sequence of the 200 nt bdf2 substrate with adenosines near the editing indicated **a-j**. **(B)** Sequence of product after 30 min incubation with hADAR2-D. Adenosine **a-j** are indicated. Methods for sequencing were performed as described in Eifler et al. (1).

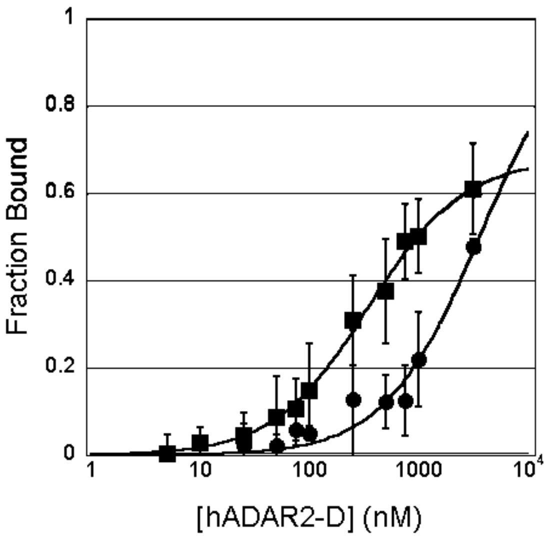

Supplementary Figure 3: Plots of fraction RNA bound determined by EMSA of ADAR2-D with substrate (**c**). Plot of fraction bound as a function of hADAR2-D concentration. ■ = (**c**) containing 8-azaN, ●= (**c**) containing A.

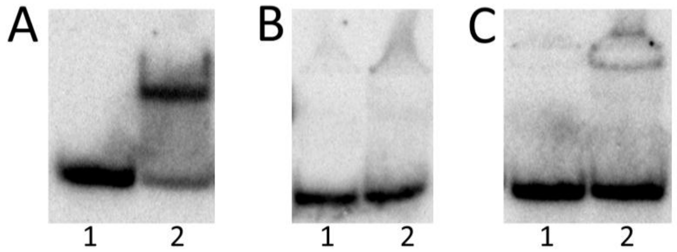

Supplementary Figure 4: (A) Autoradiogram of gel used to resolve bound from free RNA in EMSA with hADAR2-D and (**d**). **(B)** Autoradiogram of gel used to resolve bound from free RNA in EMSA with hADAR2-D and the 22 nt 8-azaN containing top strand with a DNA complement. **(C)** Autoradiogram of gel used to resolve bound from free RNA in EMSA with hADAR2-D and single stranded 22 nt 8-azaN containing top strand. Samples were equilibrated in 20 mM Tris-HCl, pH 7, 4 % glycerol, 0.5 mM DTT, 60 mM KCl, 20 mM NaCl, 0.1 mM 2-mercaptoethanol, 1.5 mM EDTA, 0.003% NP-40, 160 units/mL RNasin, 100ug/mL BSA and 1.0 µg/mL yeast tRNA for 30 min at 30 °C. 1 = 0 nM ADAR2-D, 2 = 250 nM ADAR2-D.

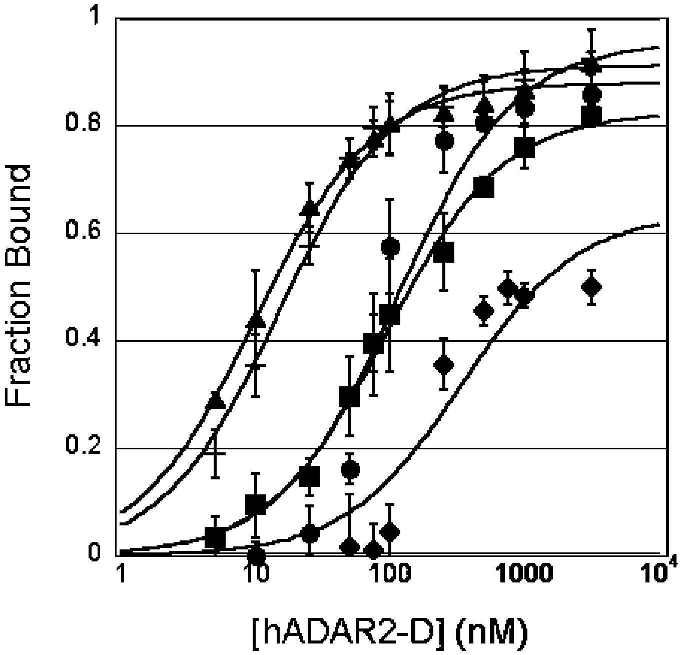

Supplementary Figure 5: Plots of fraction RNA bound determined by EMSA of ADAR2-D with substrates (**f**)-(**j**). Plot of fraction bound as a function of hADAR2-D concentration. ● = (**f**), ■ = (**g**), ◊ = (**h**), + = (**i**), ▲ = (**j**).

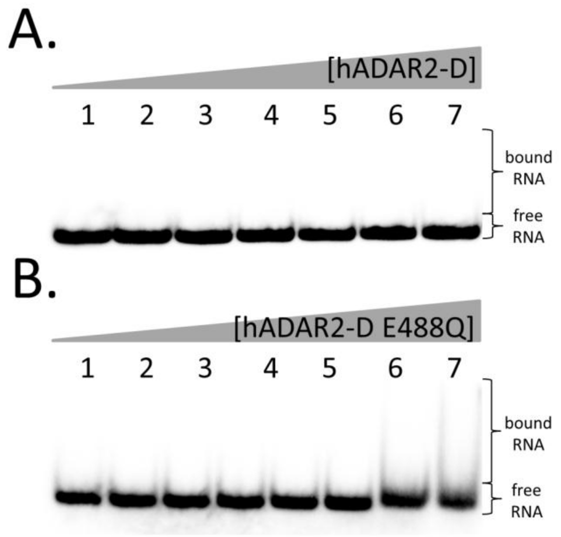

Supplementary Figure 6: (A) Autoradiogram of gel used to resolve bound from free RNA in EMSA with hADAR2-D and **(i)** where X = A. Lanes 1-7: 0, 25, 50, 75, 100, 250, 500 nM ADAR2 added. **(B)** Autoradiogram of gel used to resolve bound from free RNA in EMSA with hADAR2-D E488Q and **(i)** where X = A. Lanes 1-7: 0, 25, 50, 75, 100, 250, 500 nM hADAR2-D E488Q added.

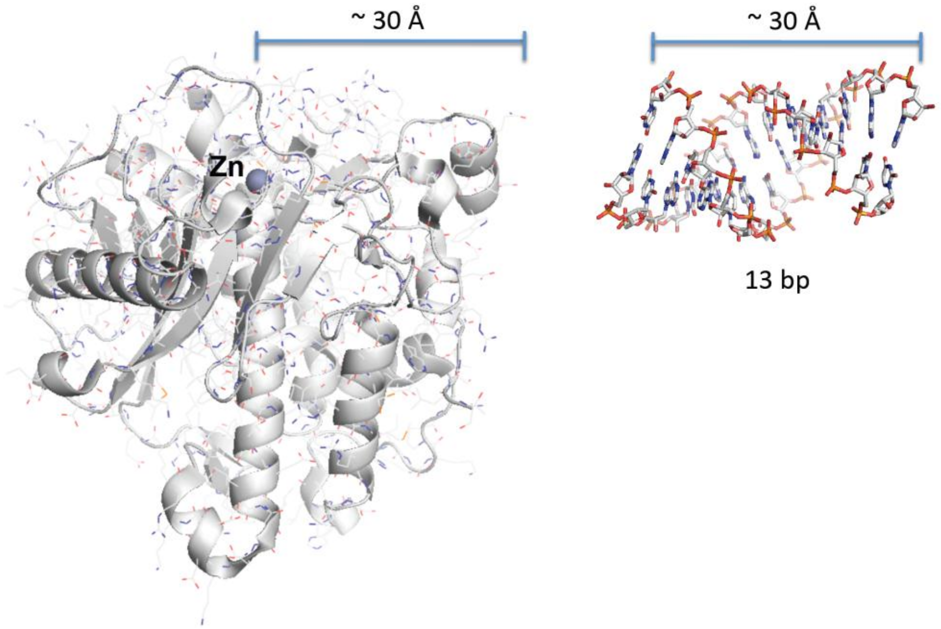

Supplementary Figure 7: hADAR-D structure and indicated distance from the zinc site to the protein's edge on the likely RNA binding surface (2). This distance corresponds to 13 bp of A-form helix at the furthest point.

Reference:

1. Eifler, T., Pokharel, S. and Beal, P.A. (2013) RNA-Seq Analysis Identifies a Novel Set of Editing Substrates for Human ADAR2 Present in Saccharomyces cerevisiae. *Biochemistry*, **52**, 7857-7869.
2. Macbeth, M.R., Schubert, H.L., VanDemark, A.P., Lingam, A.T., Hill, C.P. and Bass, B.L. (2005) Inositol hexakisphosphate is bound in the ADAR2 core and required for RNA editing. *Science*, **309**, 1534-1539.
